# How to construct and deliver an elevator pitch: a formula for the research scientist

**DOI:** 10.1186/s12919-024-00312-2

**Published:** 2024-11-26

**Authors:** Leslie A. Caromile, Ankita Jha, Jaye C. Gardiner, Ozlem Dilek, Ryoma Ohi, Lee Ligon

**Affiliations:** 1 Center for Vascular Biology, UConn Health, Farmington, CT 06030 USA; 2 National Heart, Lung, and Blood Institute, National Institutes of Health, Bethesda, MD 20814 USA; 3 Cancer Signaling and Microenvironment, Marvin & Concetta Greenberg Pancreatic Cancer Institute, Fox Chase Cancer Center, Philadelphia, PA 19111 USA; 4 Department of Chemistry and Biochemistry, College of Medical Science, George Mason University, Manassas, VA 20109 USA; 5 Department of Cell and Developmental Biology, University of Michigan, Ann Arbor, MI 48108 USA; 6 Department of Biological Sciences, Rensselaer Polytechnic Institute, Troy, NY 12180 USA

## Introduction

Scientists, especially those in the initial stages of their careers, often need help articulating their research's critical concepts and philosophies while simultaneously conveying the big picture or global problem their research addresses. In recent years, research scientists have increasingly adopted the elevator pitch as a highly effective means of quickly engaging with an audience, establishing a connection, and introducing themselves and their research interests. The elevator pitch is named after the brief ride in an elevator that usually lasts less than a minute. Initially an exclusive tool of the business world, the elevator pitch has since gained popularity among scientists for its concise format and ability to convey complex ideas succinctly, making it an invaluable component of any professional's toolkit.

While the elevator pitch idea may be familiar, its origins are debated. One account suggests that the elevator pitch began at the 1854 World’s Fair in New York City [1], where showman PT Barnum and Elisa Otis developed a plan to demonstrate the practicality of their new “elevator” invention. Regardless of their origin, elevator pitches are a powerful tool, allowing the listener to build a memorable association between them and their topic. Elevator pitches can be used in different professional environments (in person or virtual), such as during an interview or while attending a scientific conference. They can also be used with various audiences, such as potential employers, collaborators, exhibitors, colleagues, or non-scientists. Elevator pitches serve as an effective networking tool, fostering communication and providing a platform for promoting scientific research. Furthermore, explaining science quickly and efficiently gives non-scientists access to research, potentially improving public perceptions of science and scientists [2].

There are many different approaches to science communication [3]. While hundreds of websites offer courses, videos, and books that teach the art and valuable skills of crafting and delivering a persuasive elevator pitch [4, 5], only some focus on the scientist. Further, there are few resources in peer-reviewed biomedical scientific journals addressing this topic, thus leaving scientists at a disadvantage in refining this critical skill [6]. Here, we explore the purpose of a scientific elevator pitch, lay out step-by-step guidelines on developing and constructing them, and provide tips and pitfalls to consider while delivering the pitch. Additionally, we provide additional resources, and an example of a successful elevator pitch presented by Fellows who participated in the NIH/NIGMS Innovative Programs to Enhance Research Training funded Accelerating Career Transitions (ACT) program, a professional development program established and managed by the American Society for Cell Biology (ASCB).

## Developing an elevator pitch

When constructing an effective elevator pitch, it is imperative that one clearly establishes their purpose, thoroughly analyzes the intended audience, and remains cognizant of the situational context. Once a compelling pitch has been crafted for a specific scenario, it can be readily adapted to accommodate varying target audiences. In this section, we will delve into these three crucial components in-depth, offering insight and guidance to empower professionals in the scientific field to create a polished and impactful elevator pitch that effectively conveys their message.

### Identify one’s purpose

**Fig. 1:**
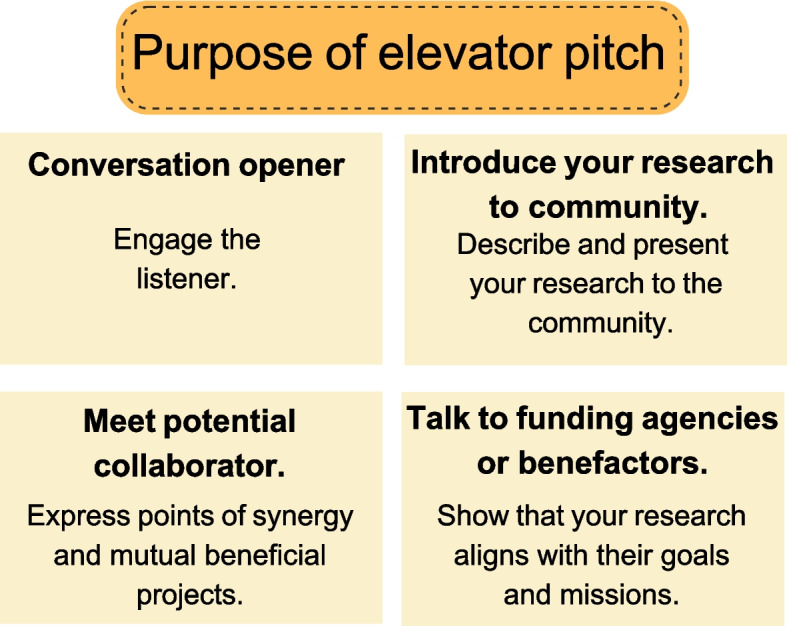
This figure addresses the common purpose of scientific elevator pitches. For scientists, elevator pitches can function as a conversation opener, help talk to the research community, meet a potential collaborator, and talk to funding agencies and benefactors

The reason scientists engage in science communication is highly varied [7]. Clarifying the purpose of an elevator pitch is an active exercise that helps identify which information should be included. Figure 1 highlights four everyday purposes of an elevator pitch, the goal, and what to include. These are 1) to open a conversation at a research conference or a networking fair, 2) to introduce oneself to a potential scientific collaborator, 3) to introduce one’s research to a broader scientific or non-scientific audience, and 4) to introduce one’s research to a funding agency or benefactor. When initiating a conversation at a research conference or a networking fair, one wants to grab the listener's attention, indicate the purpose of the conversation, and structure the conversation to lead to a follow-up discussion. When the purpose is to introduce oneself to a potential scientific collaborator, it is imperative to identify points of synergy and an outlook for why a collaboration would be mutually beneficial. In addition, it is essential to provide contact information for further discussions and a general plan for how a collaboration would work at a logistical level. When introducing research to broader and more diverse audiences, one wants to provide relevant context and a description of their research. Finally, when introducing research to a funding agency or benefactor, it is critical to clarify how the research solves problems that the benefactors aim to alleviate. It is also important to include how the research aligns with the funding agency or benefactors’ values and provide a contact for additional dialogue.

### Analyze one’s audience

**Fig. 2:**
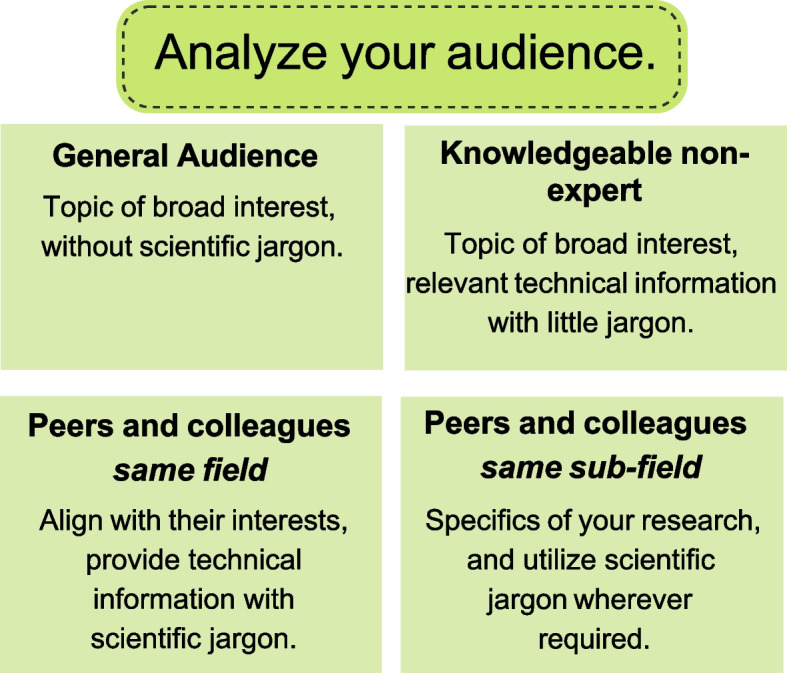
The following figure illustrates four different audiences that elevator pitches should target based on their technical knowledge of your subject matter. These groups can be broadly classified as general audiences (non-experts), educated non-experts, scientific colleagues, and peers in the same field and sub-field

Drafting and delivering an elevator pitch can be daunting, especially when one wants to engage, connect, and resonate with the audience. To achieve this, it is important to consider the audience's technical knowledge level. For scientists, audiences can be categorized into four groups based on their familiarity with the subject matter (Fig. 2). These categories include individuals who have no technical background in the field, non-experts who may have some educational background in the field, peers or colleagues working in the same field but not in the specific topic or sub-field, and peers or colleagues working in the same sub-field as the speaker. It is worth noting that these categories do not indicate an individual's overall experience or education level but rather their understanding of a particular topic. When crafting an elevator pitch, formulating questions that will help gauge the audience's familiarity with the subject matter can be considered. This approach identifies the audience's knowledge level and encourages engagement and active participation. Ultimately, the most successful elevator pitches make the audience feel respected, engaged, and connected [8, 9], which is why it is crucial to consider the audience's technical knowledge level when preparing a pitch.

When communicating with someone who has a non-technical background, it is important to establish a relevant context. This can be achieved by introducing the research within a larger, global problem framework or relating it to an analogous situation or concept. When doing so, one should make sure to avoid using scientific jargon. If one is speaking with an educated non-expert, they should connect their research to a broader scientific topic. While scientific jargon can be used in this situation, it should be limited to standard terms that are commonly used. If one is introducing their work to research professionals not in their field, it is essential to clarify how their research addresses a significant scientific concern by focusing on a specific facet of their field. Finally, when addressing a peer or colleague who conducts research within the same sub-field, there is no need to provide general scientific background information. Instead, they should highlight the exact nature of their work.

### Be aware of the context

**Fig. 3:**
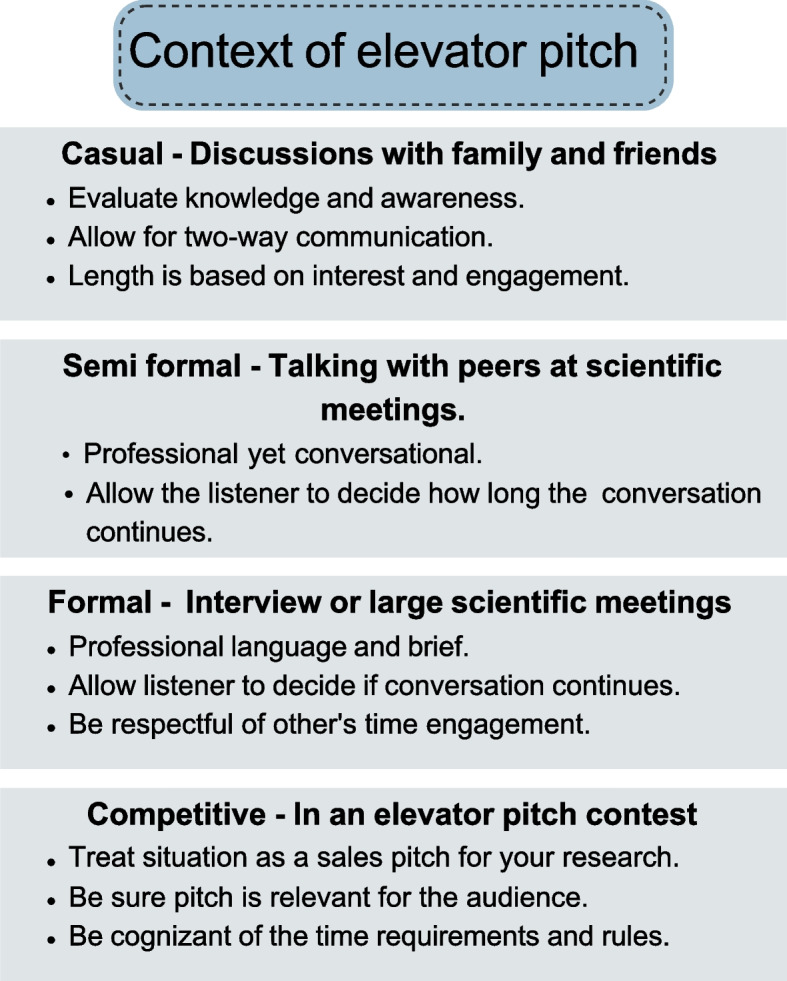
The figure illustrates that an elevator pitch's context can be tailored for different audiences, such as casual, semi-formal, formal, and competitive

To make one's pitch memorable, it is essential to understand the context of their elevator pitch, including the social and cultural atmosphere and the demands on the audience's attention [10]. Scientists are often asked to explain their work in four different contexts, including casual discussions with family or friends, semi-formal situations like talking with a speaker after their scientific conference presentation, formal events such as an interview or pitching their research to funding agencies, and elevator pitch competitions (Fig. 3). In casual situations, it is essential to gauge the knowledge and interest of the audience to allow for a two-way conversation. The pitch length should depend on the audience's interest and engagement. For semi-formal circumstances, keep the conversation professional yet casual and allow the listener to decide how long the discussion should continue. For formal situations, keep the pitch brief and use formal language to show that the speaker respects the listener's time constraints and other commitments. This will enable the listener to decide the length of the conversation. Lastly, when participating in an elevator pitch competition, treat the situation as a sales pitch for their research, expertise, and capabilities. Ensure the pitch appeals to the audience and strictly adheres to time constraints.

## Constructing and delivering the elevator pitch

Upon establishing the elevator pitch's purpose, audience, and context, the next step is to craft the pitch itself. The pitch includes three principal elements: introduction, research, and follow-up. Here, we will demonstrate how these three components intertwine and provide several key points to consider during delivery. It is important to bear in mind that the introduction serves as the first impression of the pitch and should thus be well-crafted. The research component should be thorough and relevant to the audience's interests and needs. Finally, the follow-up segment should conclude the pitch with a clear call to action, leaving a lasting impression on the audience. With these components in mind, delivering the pitch confidently and clearly is imperative. This requires practice, refinement, and understanding of the audience's perspective. By following these guidelines, the elevator pitch can become a tool for effectively communicating ideas, generating interest, and establishing rapport.

### Constructing the elevator pitch

#### Introduction and identification of target audience

The opening section of any presentation plays a crucial role in setting the tone for the entire talk. It is the presenter's opportunity to provide an introduction that will engage the audience and create a connection with them. This section should begin with an introduction of the presenter, including their full name and professional affiliation. The presenter should also make sure to greet the audience politely and respectfully. In addition to introducing themselves, the presenter should introduce a scientific problem that is either broad enough to resonate with the audience or specific enough to relate to their field of expertise. This problem should be scientifically relevant, timely, and compelling. The presenter should be able to communicate the importance of the problem and why it is worth solving. If the audience is not from a scientific background, the presenter should consider using examples, analogies, visual aids, or statistics to help explain the problem or issue. This can help the audience better understand the presenter's work and why it is important. Ultimately, the opening section of the presentation should be crafted to engage the audience and set the stage for the rest of the talk.

#### Significance of scientific research

In this section, the speaker has the opportunity to provide a comprehensive or specific overview of a particular aspect of their work. It is essential for them to carefully consider what they want their audience to take away from their research and craft their description accordingly. To effectively communicate the significance of their work, it is crucial for them to highlight how their research can address a more significant scientific problem. In addition, they can engage their audience by including audience-specific details about their approach to tackling this problem, the insights they have gained, and the potential impact of their work on the field. Doing so can create a lasting impression and positively impact the audience.

#### Conclusion and follow-up

It is recommended that the conclusion of a pitch contain an open-ended question to enhance engagement between the presenter and the audience. For maximum impact, it is crucial to select a question that is connected to one's particular area of research and underlines its significance. This can be achieved by exploring different methodologies for addressing a specific research question or by outlining the potential benefits of a collaborative effort. Furthermore, it is imperative to provide one's contact information to facilitate ongoing communication and the possibility of future collaborations.

**Fig. 4:**
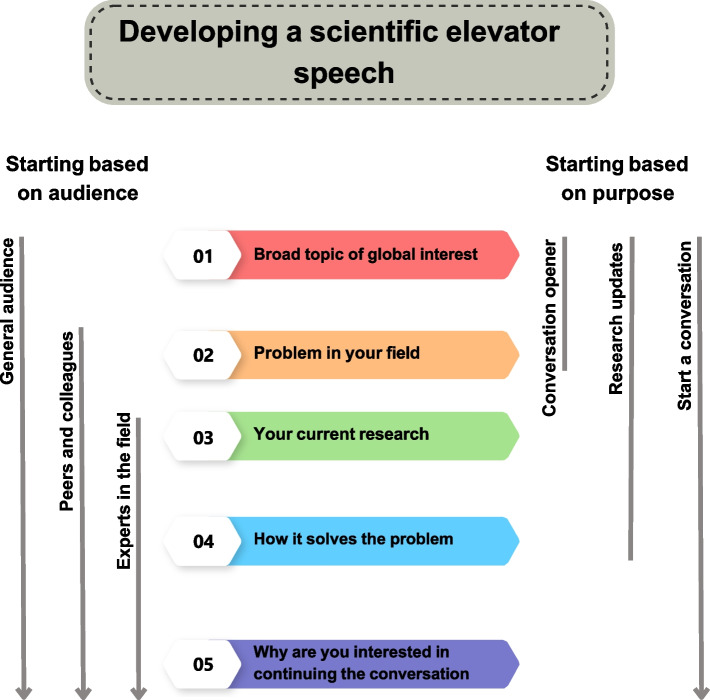
The figure represents a combination of all the elements involved in creating a scientific elevator pitch. The pitch should consist of five main points that can be adapted depending on the audience and the intended purpose. These points can also be modified according to the context in which the pitch is being presented

Figure 4 presents a comprehensive process for integrating the essential elements of a pitch, starting with the identification of the target audience and culminating in the definition of the pitch's objective. To optimize the pitch's impact, it is imperative to focus on critical concepts, employ simple language devoid of jargon, and restrict the content associated with each box to 1–2 sentences. By adhering to these guidelines, the pitch will be more approachable and comprehensible to the audience.

### Delivering the elevator pitch

The delivery of an elevator pitch is as important as its content. This segment highlights several significant concepts to consider when preparing to deliver your pitch, such as storytelling, body language, and rehearsing your pitch to ensure its naturalness. Incorporating storytelling techniques, mindful body language, and practice can help you deliver a compelling pitch that resonates with your audience.

#### Storytelling

When delivering an elevator pitch, the speaker has a mere 60 s or less to effectively convey the essence of their work. To this end, it is imperative that the speaker is able to communicate their passion for their work in a manner that inspires interest and engagement from their audience. To begin with, the art of storytelling is a fundamental aspect of delivering a compelling pitch. A well-crafted story that resonates with the audience can make a pitch more memorable. In the context of scientific storytelling, one effective strategy is to focus explicitly on the 'why' that underpins the work. The speaker can create a powerful narrative that establishes a tangible link between their research and a more significant global problem by highlighting the underlying motivation and driving forces behind the work. Ultimately, the goal is to ensure the audience remembers and associates the work with the speaker. In short, by adopting a focused, passionate, and engaging approach to elevator pitches, speakers can effectively communicate the value and significance of their work to a wide range of audiences.

#### Body language

The significance of body language in communication cannot be overstated. Research indicates that nonverbal cues conveyed through body language make up an estimated 60–65% of communication [11]. As such, when delivering an elevator pitch, it is critical to present oneself favorably. Employing good posture, maintaining eye contact, and using appropriate gestures can have a positive impact on one's audience [12–14]. Avoiding the crossing of arms and legs or any behavior that makes one appear smaller is also essential, as it not only reduces confidence but also diminishes one's perception of authority. While hand gestures can be highly effective in delivering an elevator pitch, they should be purposeful, avoiding excessive waving or extraneous movements. Additionally, any props utilized should not detract from the intended message, requiring familiarity with them beforehand to ensure a comfortable and effective demonstration [15, 16]. While exuding passion for the subject matter is desirable, it is prudent to maintain an appropriate level of enthusiasm. Finally, for online settings, ensuring the camera is appropriately angled, with ample lighting and minimal distractions, is imperative to maintain a professional appearance.

#### Practice

Elevator pitch presentations are intended to be heard rather than read. Prior to presenting, rehearsing the pitch can help to identify potential issues and refine the delivery, resulting in a more polished and natural-sounding presentation. This, in turn, can enhance confidence and enable a more effective delivery. Since natural conversations often involve varied volume, energy, and speed, adjusting the pitch's delivery can help emphasize the key concepts one wants the listener to retain. It is important to bear in mind that the elevator pitch serves as an entryway for further dialogue, and despite its brevity, it is crucial to pause after mentioning crucial points to allow the audience to ask questions. Furthermore, soliciting feedback from others can be beneficial in identifying the strengths and areas for improvement of the pitch.

## Resources and conclusion

Scientists, particularly those in the early stages of their careers, often require assistance articulating their research's fundamental concepts and philosophies while simultaneously communicating the broader picture or global issue their research seeks to address. Elevator pitches are an extremely effective method for scientists of all career stages to share their research with others and establish long-term and professionally essential connections. By providing clarity in the language used to articulate their research, these early-career scientists can better demonstrate the significance of their work and engage with their audience, thereby enabling them to effectively communicate their research to their peers, stakeholders, and the broader scientific community. However, this type of professional communication does not come naturally; it is a skill that can be learned. This need for precise communication can only be met through a collaborative effort involving the scientific community, educators, and mentors [17].

As a scientific society, the American Society for Cell Biology (ASCB) acknowledges the pivotal role played by scientific societies in providing stability to scientists during their transition across various career stages and institutions. For example, and pertinent to this manuscript, in 2012, the ASCB Public Information Committee established the ASCB Elevator Speech Contest. This contest takes place at the ASCB annual meeting and aims to encourage young researchers to be prepared to use their style and non-technical language to explain their work to anyone [18]. Additionally, the ASCB Public Policy Committee has developed the Advocacy Toolbox to help research scientists of all levels improve their scientific advocacy and communication skill sets through education and professional development sessions at the ASCB annual meeting [19].

**Fig. 5:**
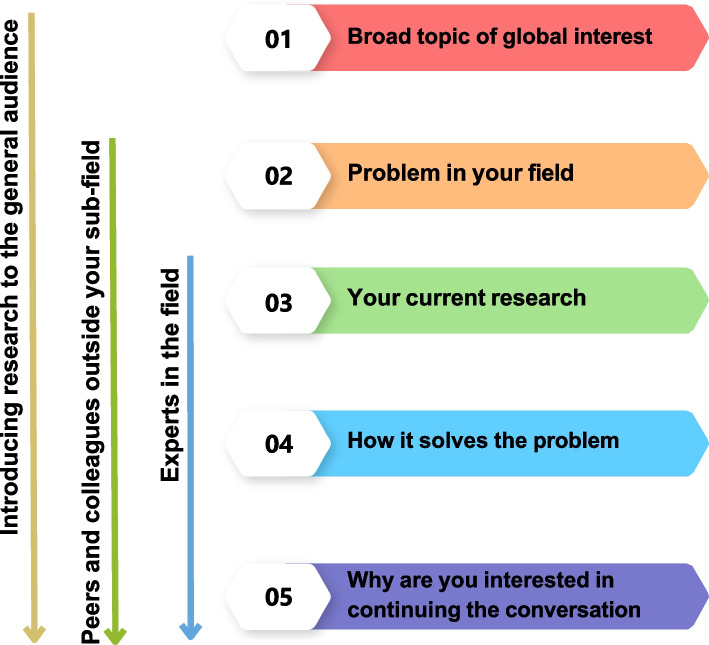
The figure demonstrates how the major components of a scientific elevator speech can be modified for different audiences and purposes

**Fig. 6:**
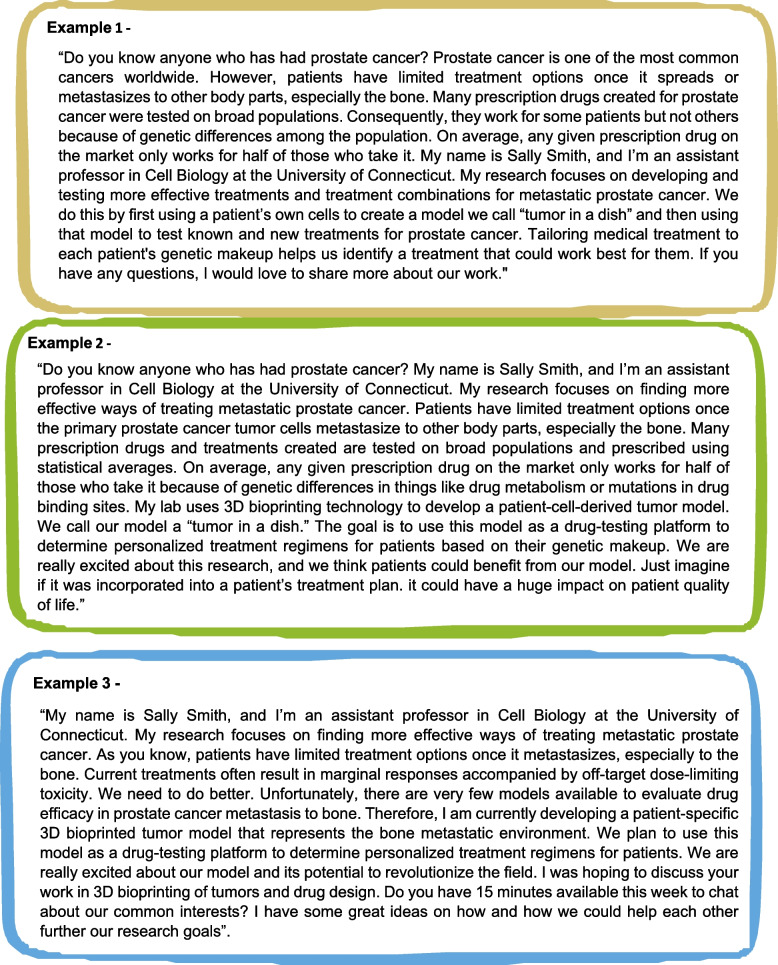
Examples of elevator pitches developed by ACT Fellows

Academic institutions have implemented various strategies to retain underrepresented (UR) scientists to promote greater diversity and inclusion. These strategies include transforming institutional culture to foster inclusivity and providing support programs that offer training and mentoring to UR scientists. The ASCB has recognized this demand and has made a solid commitment to professional development and education for UR scientists. With the help of NIH/NIGMS Innovative Programs to Enhance Research Training (IPERT) (R25) supported activities, ASCB has become a model of how a society can have a lasting impact on diversity by adapting best practices in academia to the needs and strengths of a scientific society. The Accelerating Career Transitions (ACT) program is an example of one of these IPERT-supported programs. ACT was a two-year-long, longitudinal, cohort-based professional development program for UR postdocs and assistant professors in the biological/biomedical sciences interested in transitioning into faculty and research roles. Through a summer workshop, online training, ASCB Annual meeting activities, and a permanent virtual community, ACT honed strategies for navigating critical career challenges through training in active career management and entrepreneurship skills, and research approaches. ACT also provides opportunities to practice new skills through hands-on career management and entrepreneurship practicums. As well as increased trainees’ sense of belonging and expanded their support networks through cohort-based, near-peer and peer-mentoring. One component of the ACT program focused on important professional development topics, such as creating a successful and engaging elevator pitch. Figures 5 and 6 provide examples of elevator pitches developed by ACT fellows and how these pitches can be modified for different audiences and purposes.

In conclusion, crafting an effective elevator pitch can be a valuable tool for making a strong first impression in a business or academic setting. This brief speech is designed to highlight a scientist's professional background, areas of expertise, and unique scientific capabilities. To develop a powerful and memorable elevator pitch, it is crucial to have a deep understanding of oneself and the factors that have shaped one's character and career trajectory, in addition to a keen awareness of professional goals. By following the guidelines presented here, scientists can create an elevator pitch that reflects their unique identity and leaves a lasting impression on the audience. An elevator pitch can be an essential asset in today's fast-paced, competitive environment, where the ability to communicate one's value proposition clearly and effectively is crucial to success.

## Data Availability

N/A.

## Abbreviations

ACT: Accelerating Career Transitions
ASCB: American Society for Cell Biology
NIH: National Institutes of Health
NIGMS: National Institute of General Medical Sciences
IPERT: Innovative Programs to Enhance Research Training
